# Supplementary material for: High-resolution impact-based early warning system for riverine flooding
Source: Nat Commun. 2024 May 2;15:3726. doi: 10.1038/s41467-024-48065-y (PMC11065894; doi:10.1038/s41467-024-48065-y)
Supplement: Supplementary file 1 — Supplementary Information [file 41467_2024_48065_MOESM1_ESM.pdf]

Supplementary Material for High-Resolution Impact-based Early Warning System for Riverine Flooding

Husain Najafi[1,*], Pallav Kumar Shrestha[1,2], Oldrich Rakovec[1,3], Heiko Apel[4], Sergiy Vorogushyn[4], Rohini Kumar[1], Stephan Thober[1], Bruno Merz[4,2], and Luis Samaniego[1,2,*]

[1]UFZ-Helmholtz Centre for Environmental Research, Leipzig, Germany
[2]University of Potsdam, Institute of Environmental Science and Geography, Am Neuen Palais 10, 14469 Potsdam, Germany
[3]Faculty of Environmental Sciences, Czech University of Life Sciences Prague, Praha-Suchdol 16500, Czech Republic
[4]GFZ German Research Centre for Geosciences, Section Hydrology, Potsdam, Germany
[*]Corresponding author emails: husain.najafi@ufz.de, luis.samaniego@ufz.de

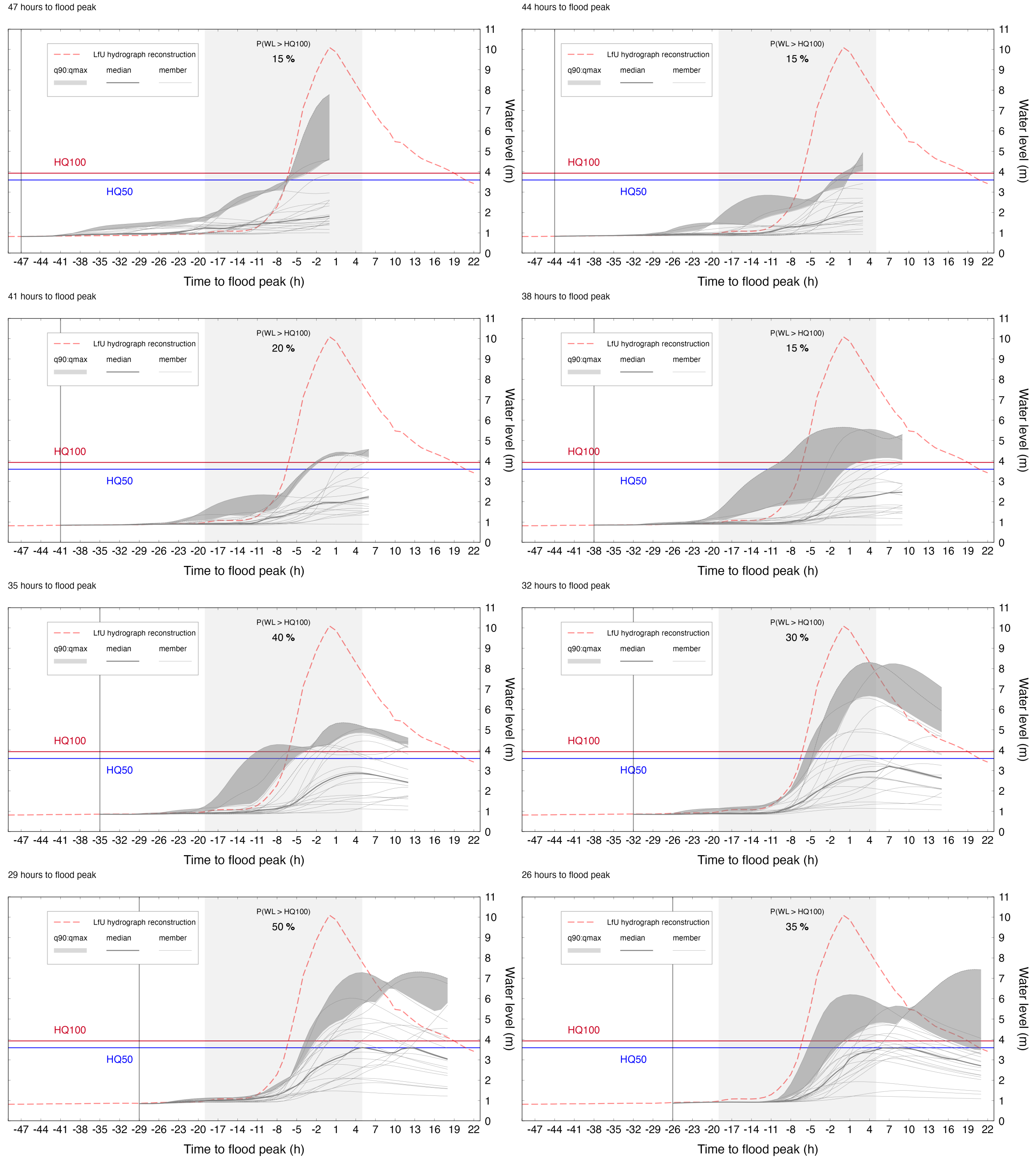

Figure S1: Ensemble water level forecasts (n=20) at gauge Altenahr for 16 initialisations between July 13, 2021 (02 CEST) and July 14, 2021 (23 CEST) based on ICON_D2_EPS - mHM. Initialization timming from 47 h to 26 h before the event. Data Sources: Observed streamflow: LfU. Observed rainfall (RADOLAN) and weather forecast (ICON_D2_EPS): Deutscher Wetterdienst.

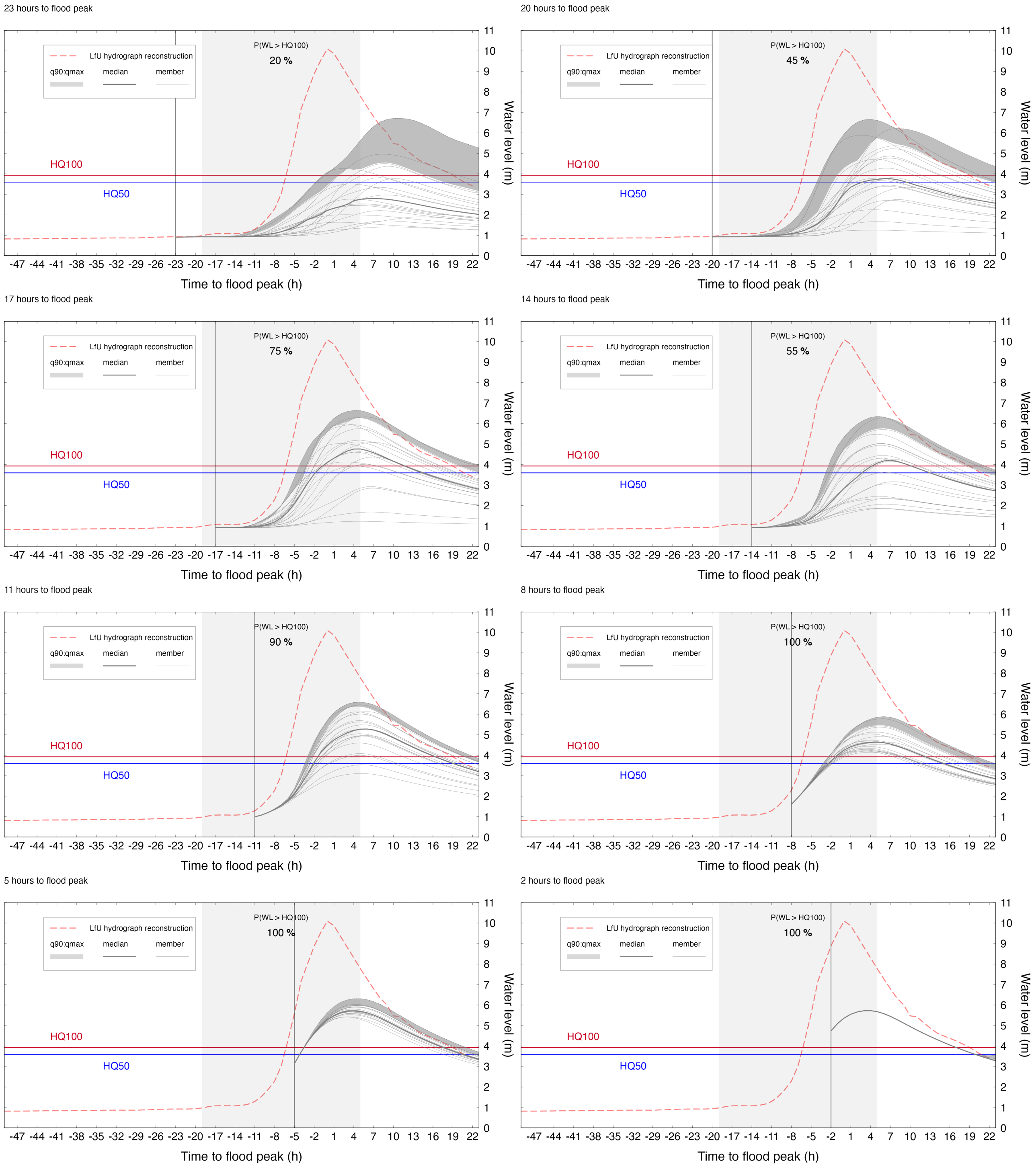

Figure S1: ... Cont. Ensemble water level forecasts. Initialization timming from 23 h to 2 h before the event.

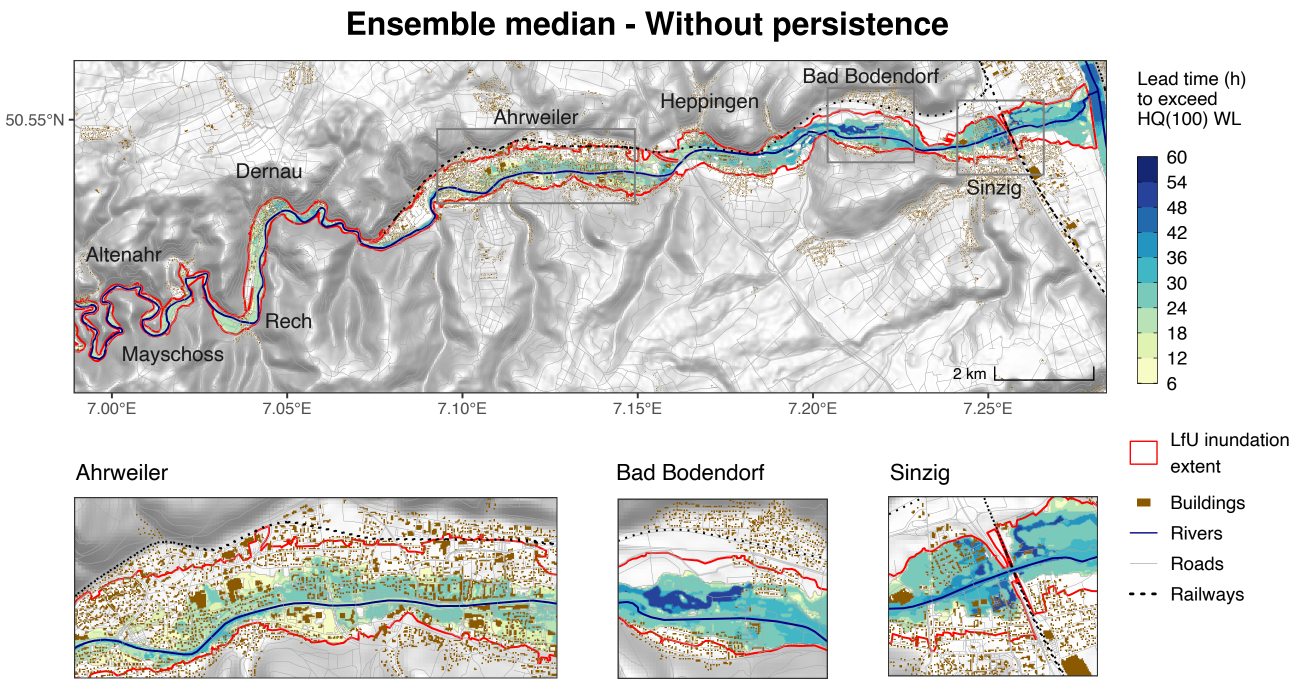

Figure S2: Raster-based lead-time map of water level downstream Altenahr gauge exceeding HQ100 levels based on ensemble median from ICON_D2_EPS-mHM-RIM2D warning chain. The red outlined areas indicate the inundation areas mapped by the State Office for the Environment (LfU) of Rheinland-Pfalz. Suplementary data sources: OSM rivers, roads and buildings: OpenStreetMap[1] contributors 2021 distributed under the Open Data Commons Open Database License (ODbL) v1.0. Hillshade: DTM[2]v0.3 (CC BY).

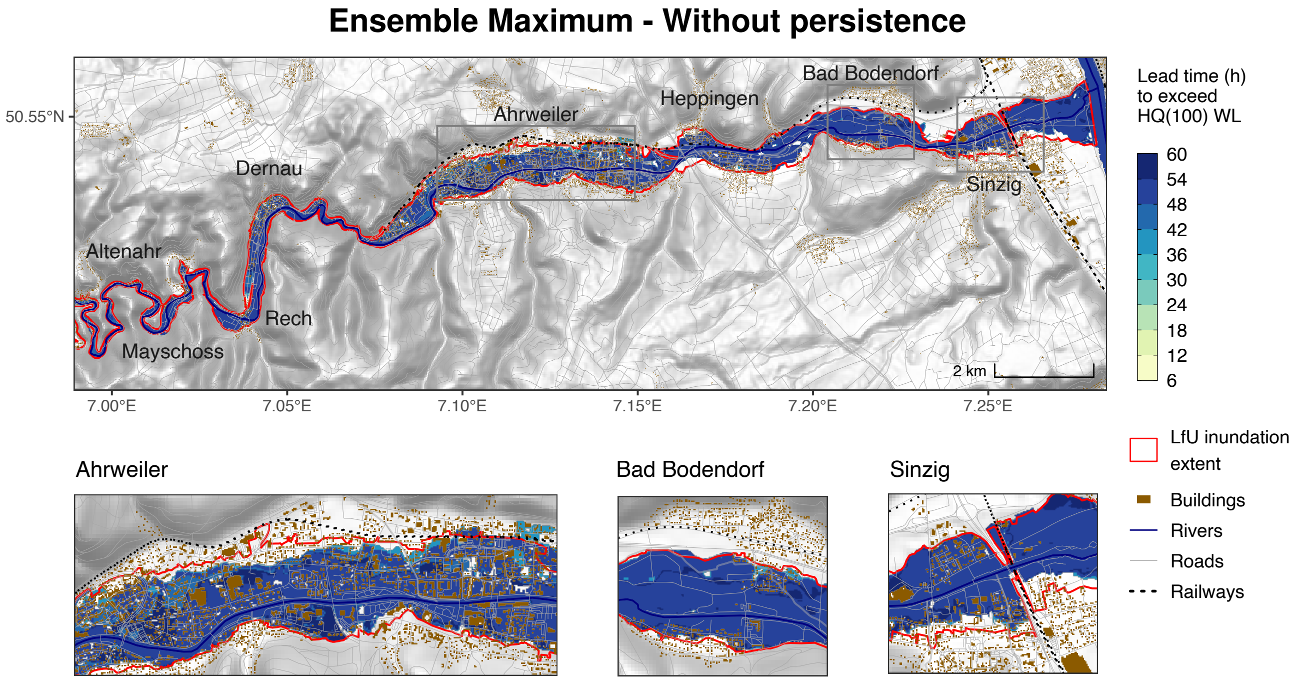

Figure S3: Raster-based lead-time map of water level downstream Altenahr gauge exceeding HQ100 levels based on (Maximum ensemble from ICON_D2_EPS-mHM-RIM2D warning chain. The red outlined areas indicate the inundation areas mapped by the State Office for the Environment (LfU) of Rheinland-Pfalz. Suplementary data sources: OSM river, roads and buildings: OpenStreetMap[1] contributors 2021 distributed under the Open Data Commons Open Database License (ODbL) v1.0. Hillshade: DTM v0.3 (CC BY)[2].

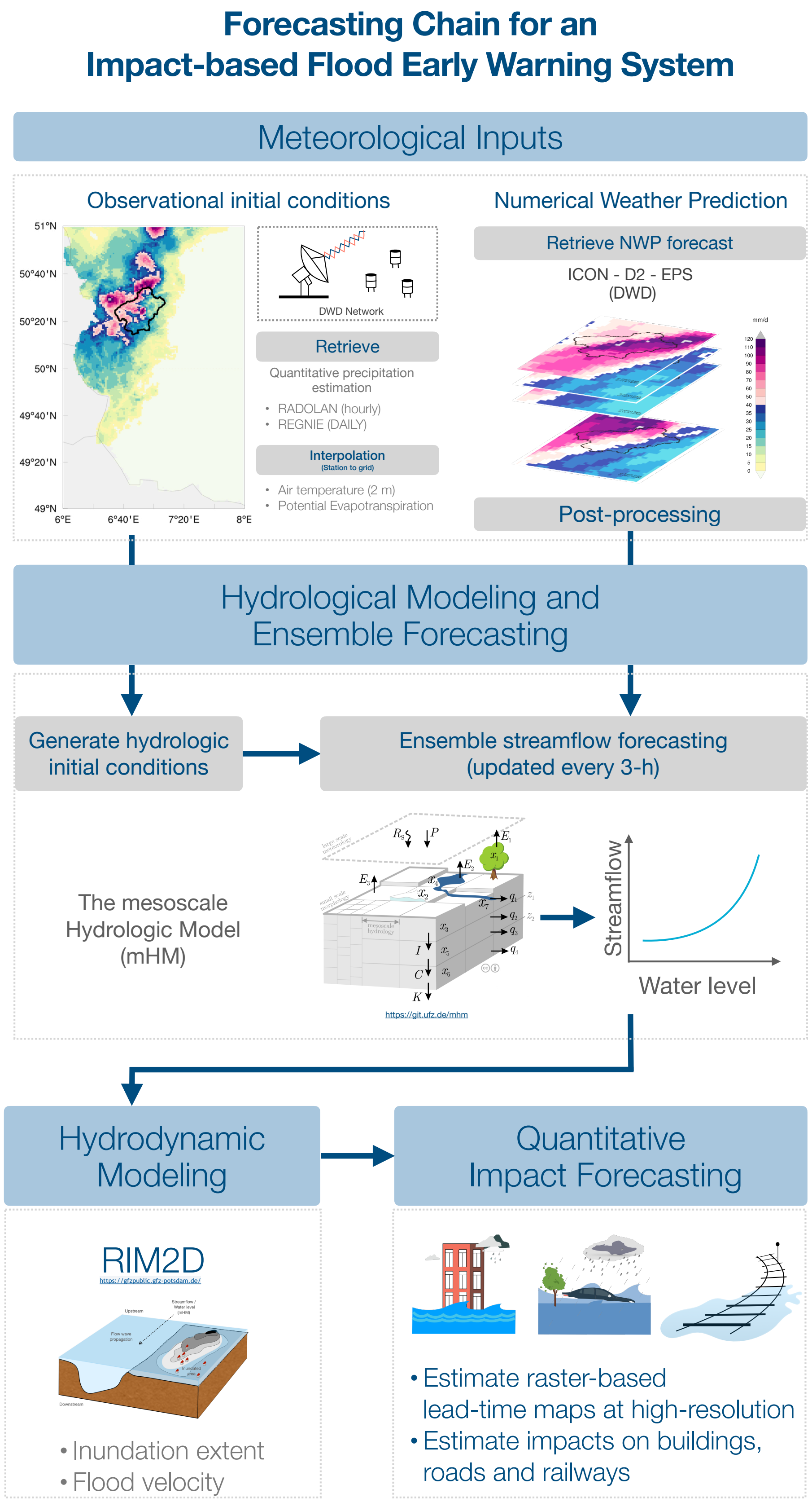

Figure S4: Schematic of the ICON_D2_EPS-mHM-RIM2D flood warning chain. Data sources: Observed rainfall fields based on RADOLAN and ICON_D2_EPS (Deutscher Wetterdienst).

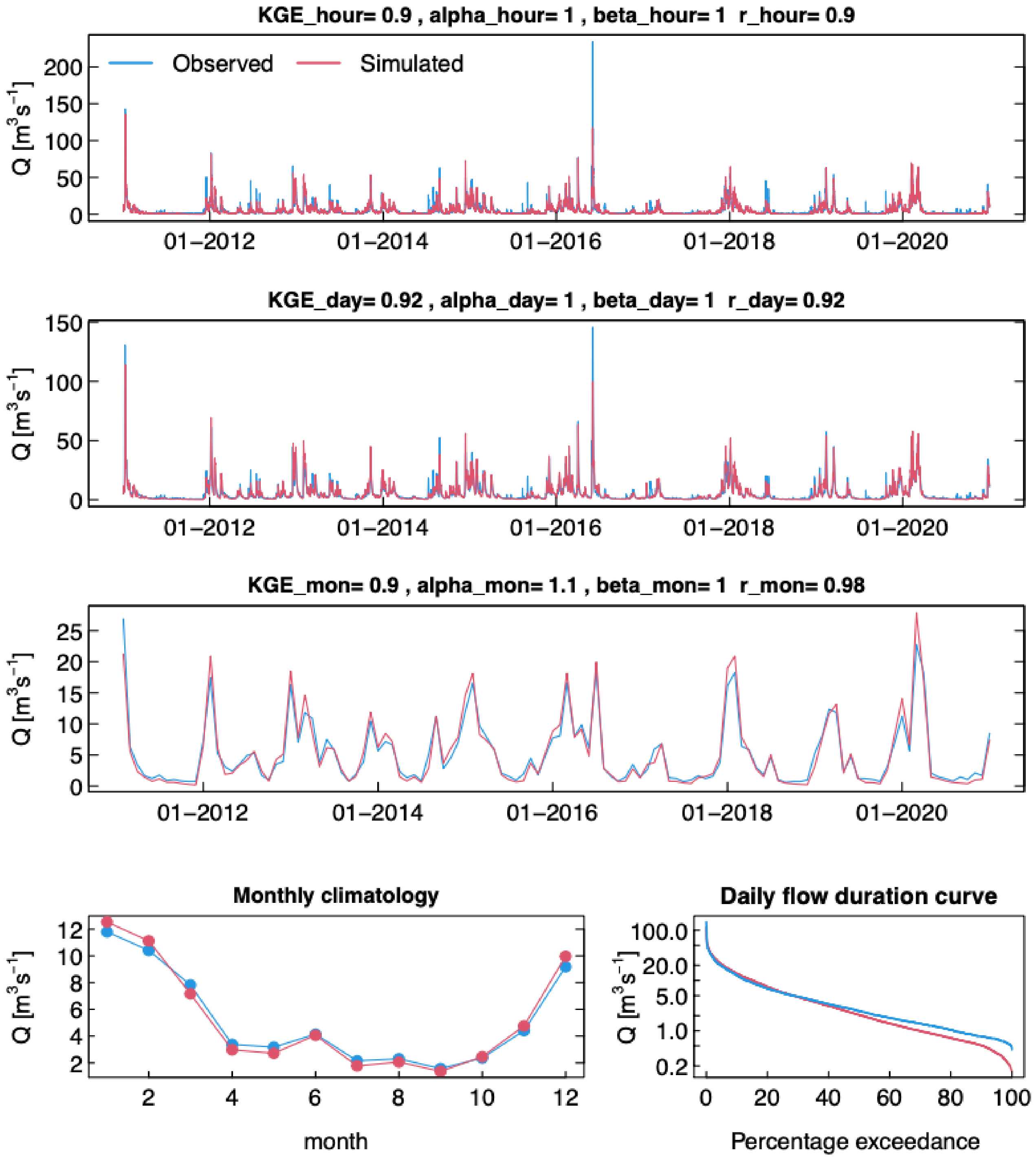

Figure S5: Time series of streamflow simulations and observation at (a) hourly, (b) daily, (c) monthly time step for the Altenahr gauge. Monthly climatology is show in panel (d) and daily flow direction curve is shown in panel (e). The respective Kling-Gupta efficiency (KGE)[3] values with an optimal value of 1 and the three components of KGE (i.e., alpha = ratio of variability, beta = ratio of bias, r = correlation) are shown in the respective panels. Data source: observed streamflow from LfU.

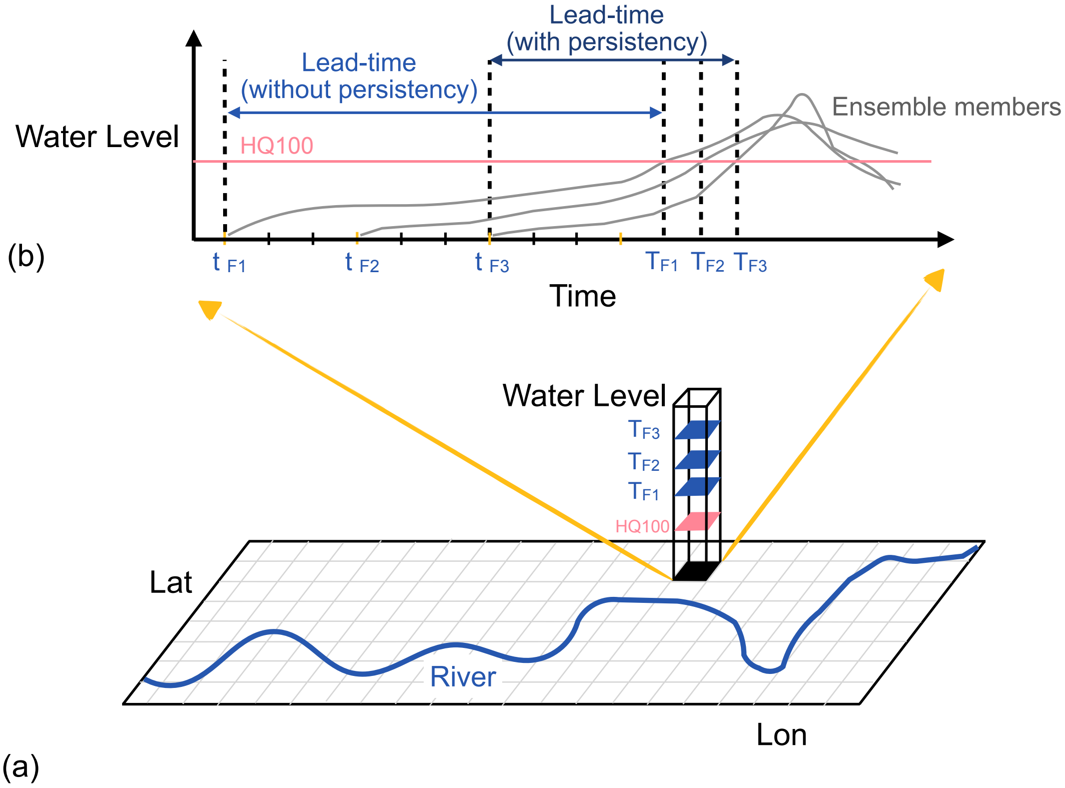

t_{Fi} : Forecast initialization for which water level exceeds HQ100 for the ith time

T_{Fi} : Forecast time step of hydrodynamic model corresponding to t_{Fi}

Figure S6: (a) Raster-based inundation forecast and water level shown for three forecast initialisations for a given pixel (b) Definition of maximum lead-time (with and without persistency) for the given pixel and an ensemble member. In defining the term lead-time with persistency, water level for three consecutive forecast initialisations exceed the HQ100.

References

1. OpenStreetMap. OpenStreetMap contributors 2021 distributed under the Open Data Commons Open DatabaseLicense (ODbL) v1.0., last access: 30 January 2022 (2022).

2. Hengl, T., Leal Parente, L., Krizan, J. & Bonannella, C. Continental Europe Digital Terrain Model at 30 m resolution based on GEDI, ICESat-2, AW3D, GLO-30, EUDEM, MERIT DEM and background layers (2021). URL `https://doi.org/10.5281/zenodo.4724549`.

3. Gupta, H. V., Kling, H., Yilmaz, K. K. & Martinez, G. F. Decomposition of the mean squared error and NSE performance criteria: Implications for improving hydrological modelling. *Journal of Hydrology* **377**, 80–91 (2009). URL `https://www.sciencedirect.com/science/article/pii/S0022169409004843`.
