# Supplementary material for: High-resolution impact-based early warning system for riverine flooding
Source: Nat Commun. 2024 May 2;15:3726. doi: 10.1038/s41467-024-48065-y (PMC11065894; doi:10.1038/s41467-024-48065-y)
Supplement: Supplementary file 3 — Reporting Summary [file 41467_2024_48065_MOESM3_ESM.pdf]

nature portfolio

Corresponding author(s): Husain Najafi, Luis Samaniego

Last updated by author(s): Mar 12, 2024

Reporting Summary

Nature Portfolio wishes to improve the reproducibility of the work that we publish. This form provides structure for consistency and transparency in reporting. For further information on Nature Portfolio policies, see our Editorial Policies and the Editorial Policy Checklist.

Statistics

For all statistical analyses, confirm that the following items are present in the figure legend, table legend, main text, or Methods section.

n/a	Confirmed	
☐	☒	The exact sample size (*n*) for each experimental group/condition, given as a discrete number and unit of measurement
☒	☐	A statement on whether measurements were taken from distinct samples or whether the same sample was measured repeatedly
☒	☐	The statistical test(s) used AND whether they are one- or two-sided<br>*Only common tests should be described solely by name; describe more complex techniques in the Methods section.*
☒	☐	A description of all covariates tested
☐	☒	A description of any assumptions or corrections, such as tests of normality and adjustment for multiple comparisons
☐	☒	A full description of the statistical parameters including central tendency (e.g. means) or other basic estimates (e.g. regression coefficient) AND variation (e.g. standard deviation) or associated estimates of uncertainty (e.g. confidence intervals)
☒	☐	For null hypothesis testing, the test statistic (e.g. *F*, *t*, *r*) with confidence intervals, effect sizes, degrees of freedom and *P* value noted<br>*Give P values as exact values whenever suitable.*
☒	☐	For Bayesian analysis, information on the choice of priors and Markov chain Monte Carlo settings
☐	☒	For hierarchical and complex designs, identification of the appropriate level for tests and full reporting of outcomes
☒	☐	Estimates of effect sizes (e.g. Cohen's *d*, Pearson's *r*), indicating how they were calculated

Our web collection on statistics for biologists contains articles on many of the points above.

Software and code

Policy information about availability of computer code

Data collection	N/A
Data analysis	REGNIE and RADOLAN data, all from DWD, are freely available for research at the Open Data Portal (https://opendata.dwd.de, last access: 9 May 2022). DWD weather forecasts (ICON-D2-EPS) are available at Pamore (PArallelMOdel data REtrieve from Oracle databases) after registration (https://www.dwd.de/EN/ourservices/pamore/pamore.html, last access: 29~Nov 2022).<br>OSM river network and buildings are available from ©OpenStreetMap contributors2022, distributed under the Open Data Commons Open Database License (ODbL) v1.0. The OpenStreetMap data are open source.<br>The Digital Elevation Model (DEM) with a 10-meter grid, provided by GeoBasis-DE/BKG in 2022, data are available under restricted access for license reason, access can be obtained by ((https://gdz.bkg.bund.de/index.php/default/digitale-geodaten/digitale-gelandemodelle/digitales-gelandemodell-gitterweite-10-m-dGm10.html)). The digital elevation model of the BKG is available upon request for non-commercial use. If you wish to obtain this DEM, it is recommended to visit the provided link and follow the contact information or procedures specified on GeoBasis-DE/BKG's website, Bundesamtfür Kartographie und Geodäsie, 2022.<br>The hourly data from the gauges in the Ahr basin data are were kindly processed and provided by Michael Göller, Landesamt für Umwelt,Rheinland-Pfalz and is available under restricted access.<br>The mHM subdaily discharge data (calibrated) and ensemble forecasts (n=20) generated for each initialization in this study have been deposited in the mHM_ensemle_water_level_forecast and output_calib_01 database under accession code [https://www.ufz.de/record/dmp/archive/14607/de/]. Streamflow gauge data and mHM output are provided for calibration of hydrological model.<br>The RIM2D output data (calibrated) generated for each initialisation in this study have been deposited in the RIM2D_WL_FORECAST database under accession code [https://www.ufz.de/record/dmp/archive/14607/de/].<br>The Flood hazard maps used in this study have been deposited in the HQ100_extent database under accession code [https://www.ufz.de/record/dmp/archive/14607/de/]. We used the flood hazard maps from Rhineland-Palatinate used show the updated hazard maps (https://

hochwassermanagement.rlp-umwelt.de/servlet/is/200041/) accessed on 23.06.2022. The flood hazard map could have been updated after the 2021 flood event. The codes and algorithms to generate figures provided in this study can be accessed under this link \url{https://www.ufz.de/record/dmp/archive/14607/de/}.

For manuscripts utilizing custom algorithms or software that are central to the research but not yet described in published literature, software must be made available to editors and reviewers. We strongly encourage code deposition in a community repository (e.g. GitHub). See the Nature Portfolio guidelines for submitting code & software for further information.

Data

Policy information about availability of data

All manuscripts must include a data availability statement. This statement should provide the following information, where applicable:

- Accession codes, unique identifiers, or web links for publicly available datasets
- A description of any restrictions on data availability
- For clinical datasets or third party data, please ensure that the statement adheres to our policy

REGNIE and RADOLAN data, all from DWD, are freely available for research at the Open Data Portal (https://opendata.dwd.de, last access: 9 May 2022). DWD weather forecasts (ICON-D2-EPS) are available at Pamore (PArallelMOdel data REtrieve from Oracle databases) after registration (https://www.dwd.de/EN/ourservices/pamore/pamore.html, last access: 29~Nov 2022).
The Digital Elevation Model (DEM) data with a 10-meter grid, provided by GeoBasis-DE/BKG in 2012, is available under restricted access for free use, access can be obtained by (https://gdz.bkg.bund.de/index.php/default/digitale-geodaten/digitale-gelandemodelle/digitales-gelandemodell-gitterweite-10-m-dGm10.html). If you wish to obtain this DEM, it is recommended to visit the provided link and follow the contact information or procedures specified on GeoBasis-DE/BKG's website.
OSM river network and buildings are available from ©OpenStreetMap contributors2017, distributed under the Open Data Commons Open Database License (ODbL) v1.0. The OpenStreetMap data are open source.
The hourly data from the gauges in the Ahr basin were kindly processed and provided by Michael Göller, Landesamt für Umwelt, Rheinland-Pfalz (https://wasserportal.rlp-umwelt.de/servlet/is/8181/).
Streamflow gauge data and mHM output are provided for calibration of hydrological model.
Flood hazard maps are based on (https://geoportal.bafg.de/karten/HWRM_Aktuell/#). We used the flood hazard maps from Rhineland-Palatinate used show the updated hazard maps (https://hochwassermanagement.rlp-umwelt.de/servlet/is/200041/) accessed on 23.06.2022. The flood hazard map could have been updated after the 2021 flood event.
The dataset for generating flood impact forecasting can be accessed under this link (https://www.ufz.de/record/dmp/archive/14607/en/).
Post event-mapping of the inundated areas of the European Summer Flood in Ahr Valley was mapped and provided by Landesamt für Umwelt (LfU) Rheinland-Pfalz. Abteilung Hydrologie h:p://www.lfu.rlp.de/ contact: Thomas.Be:mann@lfu.rlp.deThe supplementary data for Figure 2, Figure 4 and FigureS5 are provided with this paper.

Research involving human participants, their data, or biological material

Policy information about studies with human participants or human data. See also policy information about sex, gender (identity/presentation), and sexual orientation and race, ethnicity and racism.

Reporting on sex and gender	N/A
Reporting on race, ethnicity, or other socially relevant groupings	N/A
Population characteristics	N/A
Recruitment	N/A
Ethics oversight	N/A

Note that full information on the approval of the study protocol must also be provided in the manuscript.

Field-specific reporting

Please select the one below that is the best fit for your research. If you are not sure, read the appropriate sections before making your selection.

☐ Life sciences ☐ Behavioural & social sciences ☒ Ecological, evolutionary & environmental sciences

For a reference copy of the document with all sections, see nature.com/documents/nr-reporting-summary-flat.pdf

Ecological, evolutionary & environmental sciences study design

All studies must disclose on these points even when the disclosure is negative.

Study description	An ensemble hindcast experiment is designed to evaluate the predictability of the 2021 European Summer flood based on an advanced high-resolution impact-based early warning system. The aim is to show how using high-resolution impact-based early warning can support tailored decision making and advisable actions for flood preparedness. Since we used the state-of-the-art
Research sample	We have used the ensemble forecasting system ICON-D2 EPS for precipitation and water level forecasts with 20 ensemble members. For generating the ensemble members, some of the features of the forecasting system are changed. To generate the ensemble members, some of the features of the forecasting system (the lateral boundary conditions,initial state, soil moisture, and model physics) are changed by DWD.
Sampling strategy	No statistical methods/calcultiom was used to predetermine sample size from the full ensemble set for atmospheric and hydrological forecast. We have used all ensemble members (n=20) generated by the latest regional ensemble forecast model ICON-D2 EPS from the German weather Service (DWD) for each model initialisation. Since inundation modeling is computationally expensive, we have selected the ensembles corresponding to minimum, 25 percentile, median, 75 percentile and maximum to represent the uncertainty propagated from atmospheric forecast to inundation and impact forecasting. For operational purposes, selection of different ensemble ranges or additional percentiles of interest for local authorities and flood managers is possible based on their expert knowledge and available computational resources.
Data collection	Data used for this study was recorded by the German Weather Service, the Landesumweltamt Rheinland-Pfalz (LfU) and Copernicus, which is an EU programme aimed at developing European information services based on satellite Earth Observation and in situ (non space) data, OpenStreetMap and the Federal Institute for Geosciences and Natural Resources- Bundesanstalt für Geowissenschaften und Rohstoffe(BGR).
Timing and spatial scale	The atmospheric forecasts (horizontal resolution of 2.2 km) issued between July 13-14, 2021 are used for forecasting flood as the numerical weather prediction forecasts period is 48 hour. Radolan data (hourly gridded precipitation) with a spatial resolution of 1 km² are available from 2005 onward. Hydrological simulations for reference run and calibration have been conducted from 2006-2021. We considered a 10-year simulation period (1.1.2011--31.12.2020) with five years of warm-up. Hydrological model simulation of mHM is conducted in 0.01562◦ × 0.01562◦ equivalent to ?1.2km×1.2km. The spatial resolution of hydrodynamic model RIM2D (inundation modeling) and impact forecasting is 10 meter.
Data exclusions	The data of the 2021 flood was excluded from the calibration to provide analysis as a hindcast experiment. In such a way, we have provided the result to investigate which information could have been used for local authorities in quasi-real-time.
Reproducibility	All data and codes used for the study are open-source or accessible for research purposes and are reproducible.
Randomization	Not relevant
Blinding	Not relevant

Did the study involve field work? ☐ Yes ☒ No

Reporting for specific materials, systems and methods

We require information from authors about some types of materials, experimental systems and methods used in many studies. Here, indicate whether each material, system or method listed is relevant to your study. If you are not sure if a list item applies to your research, read the appropriate section before selecting a response.

Materials & experimental systems

n/a	Involved in the study
☒	☐ Antibodies
☒	☐ Eukaryotic cell lines
☒	☐ Palaeontology and archaeology
☒	☐ Animals and other organisms
☒	☐ Clinical data
☒	☐ Dual use research of concern
☒	☐ Plants

Methods

n/a	Involved in the study
☒	☐ ChIP-seq
☒	☐ Flow cytometry
☒	☐ MRI-based neuroimaging

Plants

Seed stocks	Not relevant
Novel plant genotypes	Not relevant
Authentication	Not relevant
